# Cyclooxygenase-2 contributes to oxidopamine-mediated neuronal inflammation and injury via the prostaglandin E2 receptor EP2 subtype

**DOI:** 10.1038/s41598-017-09528-z

**Published:** 2017-08-25

**Authors:** Xu Kang, Jiange Qiu, Qianqian Li, Katherine A. Bell, Yifeng Du, Da Woon Jung, Jae Yeol Lee, Jiukuan Hao, Jianxiong Jiang

**Affiliations:** 1 Division of Pharmaceutical Sciences, James L. Winkle College of Pharmacy, University of Cincinnati Academic Health Center, Cincinnati, Ohio 45267-0514 USA; 2 Department of Cell Biology and Institute of Biomedicine, College of Life Science and Technology, Jinan University, Guangzhou, Guangdong, 510632 China; 3 Research Institute for Basic Sciences and Department of Chemistry, College of Sciences, Kyung Hee University, Seoul, 02447 Republic of Korea

## Abstract

Cyclooxygenase-2 (COX-2) triggers pro-inflammatory processes that can aggravate neuronal degeneration and functional impairments in many neurological conditions, mainly via producing prostaglandin E2 (PGE_2_) that activates four membrane receptors, EP1-EP4. However, which EP receptor is the culprit of COX-2/PGE_2_-mediated neuronal inflammation and degeneration remains largely unclear and presumably depends on the insult types and responding components. Herein, we demonstrated that COX-2 was induced and showed nuclear translocation in two neuronal cell lines – mouse Neuro-2a and human SH-SY5Y – after treatment with neurotoxin 6-hydroxydopamine (6-OHDA), leading to the biosynthesis of PGE_2_ and upregulation of pro-inflammatory cytokine interleukin-1β. Inhibiting COX-2 or microsomal prostaglandin E synthase-1 suppressed the 6-OHDA-triggered PGE_2_ production in these cells. Treatment with PGE_2_ or EP2 selective agonist butaprost, but not EP4 agonist CAY10598, increased cAMP response in both cell lines. PGE_2_-initiated cAMP production in these cells was blocked by our recently developed novel selective EP2 antagonists – TG4-155 and TG6-10-1, but not by EP4 selective antagonist GW627368X. The 6-OHDA-promoted cytotoxicity was largely blocked by TG4-155, TG6-10-1 or COX-2 selective inhibitor celecoxib, but not by GW627368X. Our results suggest that PGE_2_ receptor EP2 is a key mediator of COX-2 activity-initiated cAMP signaling in Neuro-2a and SH-SY5Y cells following 6-OHDA treatment, and contributes to oxidopamine-mediated neurotoxicity.

## Introduction

Cyclooxygenase (COX) is the enzyme responsible for the rate-determining step in the synthesis of bioactive lipids – prostanoids consisting of prostaglandin D2 (PGD_2_), PGE_2_, PGF_2α_, prostacyclin PGI_2_ and thromboxane TXA_2_, and has two isoforms – COX-1 and COX-2^1, 2^. COX-1 is constitutively expressed in a wide range of tissues to maintain homeostatic prostanoids that are essential for many biological functions such as angiogenesis, vasodilatation, platelet function, tissue maintenance, etc. COX-2 is usually present at low levels under normal conditions, but is rapidly and robustly induced by stimuli including infection, injury and pain to initiate pro-inflammatory processes that could facilitate and maintain the disease states^3–5^. As a major COX-2 product within the brain, PGE_2_ has been widely thought to promote the neuronal inflammation and degeneration in many neurological diseases such as ischemic stroke^6, 7^, epilepsy^8–10^, neurodegenerative diseases^11–13^, brain tumor^14, 15^, inflammatory pain^16^, etc. PGE_2_ can bind and activate four G protein-coupled receptors (GPCRs): EP1, EP2, EP3 and EP4. The EP receptor that is directly responsible for COX-2/PGE_2_-mediated brain inflammation and injury remains elusive and is presumably dependent on the brain insult types and the responding cells and molecules^12^.

Recent studies on animal models suggest that the inflammatory PGE_2_ signaling is involved in the pathogenesis of Parkinson’s disease (PD)^17–20^, a movement disorder that usually affects the elderly and is commonly symptomized by tremor, rigidity, akinesia/bradykinesia and postural instability. The condition is caused by the progressive death of dopaminergic neurons in the substantial nigra pars compacta (SNpc), leading to irreversible destruction of the nigrostriatal pathway^21^. The molecular mechanisms underlying the loss of SNpc neurons are not fully understood, but have been linked to several chronic pathogenic processes, such as brain inflammation, oxidative stress, mitochondrial impairment, and dysfunction in proteasomal or autophagic protein degradation^21^. Organic compound 2,4,5-trihydroxyphenethylamine – more commonly known as 6-hydroxydopamine (6-OHDA) – is a neurotoxin and has been widely used to induce PD symptoms in experimental animals owing to its capability to selectively destroy dopaminergic neurons^22, 23^. As a synthetic analogue of dopamine, 6-OHDA enters the cells via dopamine specific reuptake transporters and causes progressive neuronal death through molecular mechanisms that remain largely unknown^21^.

The neuroblastoma cell lines – mouse-derived Neuro-2a and human SH-SY5Y – preserve many aspects of SNpc neurons^24–27^, and thus are commonly used as *in vitro* models to study the signaling pathways of inflammation, oxidative stress and apoptosis in dopaminergic neurons. In this study, we investigated the COX-2-associated inflammatory processes in Neuro-2a and SH-SY5Y cells following 6-OHDA insult. Taking advantage of our recently developed novel selective small-molecule antagonists, the involvement of PGE_2_ and its EP receptors in 6-OHDA-induced neuronal toxicity and inflammation was also examined.

## Results

### Neuro-2a and SH-SY5Y cells are TH positive and susceptible to 6-OHDA-mediated cytotoxicity

**Figure 1:**
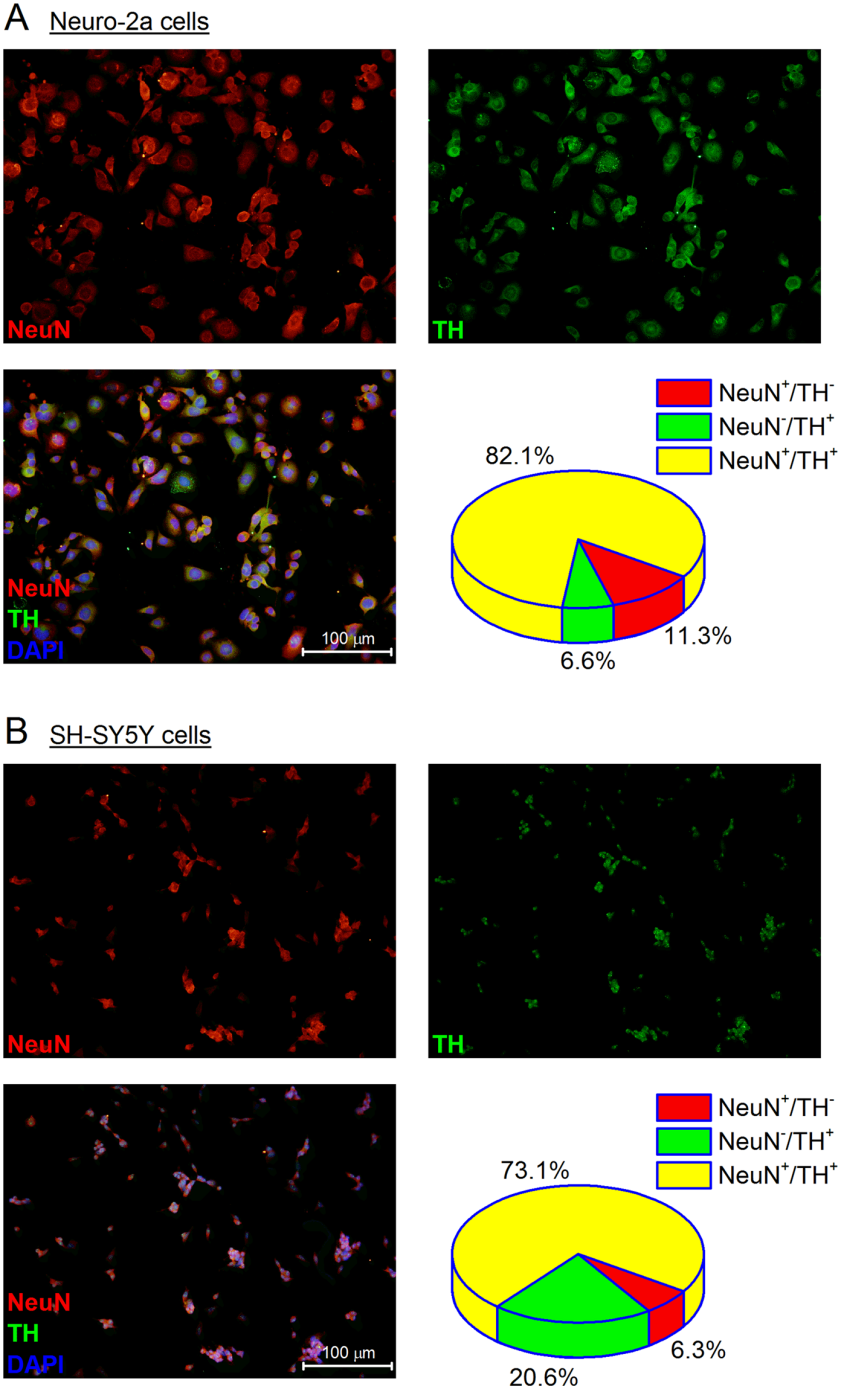
Mouse Neuro-2a and human SH-SY5Y cells are NeuN positive and express tyrosine hydroxylase (TH). Immunostaining was performed to show the expression of NeuN (red) and TH (green) in Neuro-2a cells (**A**) and SH-SY5Y cells (**B**). The cell nuclei were visualized by DAPI staining (blue) and cells were counted in 5 random fields at a magnification of 200x. Data were reported as the percentage of cells/field. Note that the majority of cells are both NeuN and TH positive. The size of SH-SY5Y cells on average is smaller than that of Neuro-2a cells, which together with their slower growth rate might explain the lower cell density of SH-SY5Y cells. Scale bar = 100 µm.

Neuro-2a is a mouse neuroblastoma cell line derived from neural crest with many features of neurons, including neurofilaments^28^; whereas SH-SY5Y is a human originated cell line that was initially isolated from a bone marrow biopsy removed from a four-year-old girl with neuroblastoma^29^. Because of their neuronal background and neuron-like properties, these two cell lines have been widely used as *in vitro* models to study neuronal function and differentiation, axonal growth, neuronal signaling, neurotoxicity, and neurodegeneration, particularly in Parkinson’s disease (PD)^30–32^. We purchased both cell lines directly from the American Type Culture Collection (ATCC), and first examined their neuronal background by immunochemistry. As shown in Fig. 1, both cultured Neuro-2a and SH-SY5Y cells widely expressed NeuN – a canonical neuronal biomarker, and tyrosine hydroxylase (TH) – the enzyme responsible for the first step of the dopamine synthesis in the SNpc neurons. In fact, the vast majority of cells – 82.1% Neuro-2a and 73.1% SH-SY5Y cells – were identified both NeuN and TH positive.

**Figure 2:**
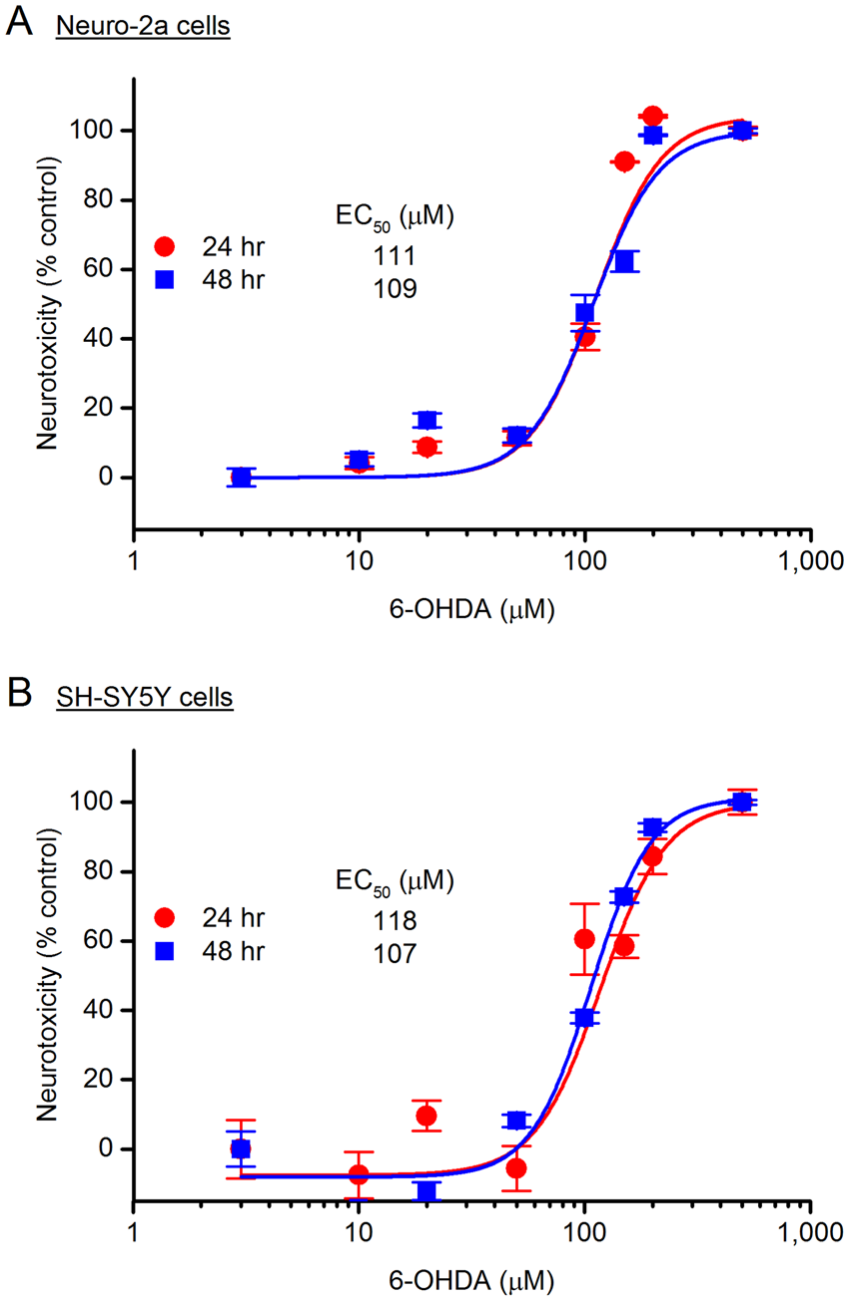
6-hydroxydopamine (6-OHDA) induces neurotoxicity. 6-OHDA caused cytotoxicity in both Neuro-2a cells (**A**) and SH-SY5Y cells (**B**) in a concentration dependent manner, shown by the 6-OHDA dose-response curves. Cells were treated with 6-OHDA at different concentrations for 24 or 48 hr, and the cell viability was measured by MTT [3-(4,5-dimethylthiazol-2-yl)-2,5-diphenyltetrazolium bromide] reduction assay. 6-OHDA EC_50_ = 111 µM for 24 hr incubation and 109 µM for 48 hr incubation in the Neuro-2a cells; 6-OHDA EC_50_ = 118 µM for 24 hr incubation and 107 µM for 48 hr incubation in the SH-SY5Y cells. Data are shown as mean ± SEM (*n* = 4–6).

As a synthetic oxidopamine, 6-OHDA is a widely used neurotoxin to bleach dopaminergic neurons and destruct the nigrostriatal pathway in rodent PD models^21–23^. Consistent with previous findings^25, 31^, 6-OHDA was shown here to decrease the viability of both Neuro-2a cells (Fig. 2A) and SH-SY5Y cells (Fig. 2B) in a concentration-dependent manner with similar EC_50_ values (~110 µM), measured by 3-(4,5-dimethylthiazol-2-yl)-2,5-diphenyltetrazolium bromide (MTT) reduction assay 24 hr after treatment. It appears that incubation for an additional day did not further increase the 6-OHDA-mediated cytotoxicity in these two cell lines (Fig. 2A and B); therefore, we used up to 24 hr treatment for 6-OHDA throughout the rest of this study.

### 6-OHDA induces COX-2 expression and nuclear translocation

**Figure 3:**
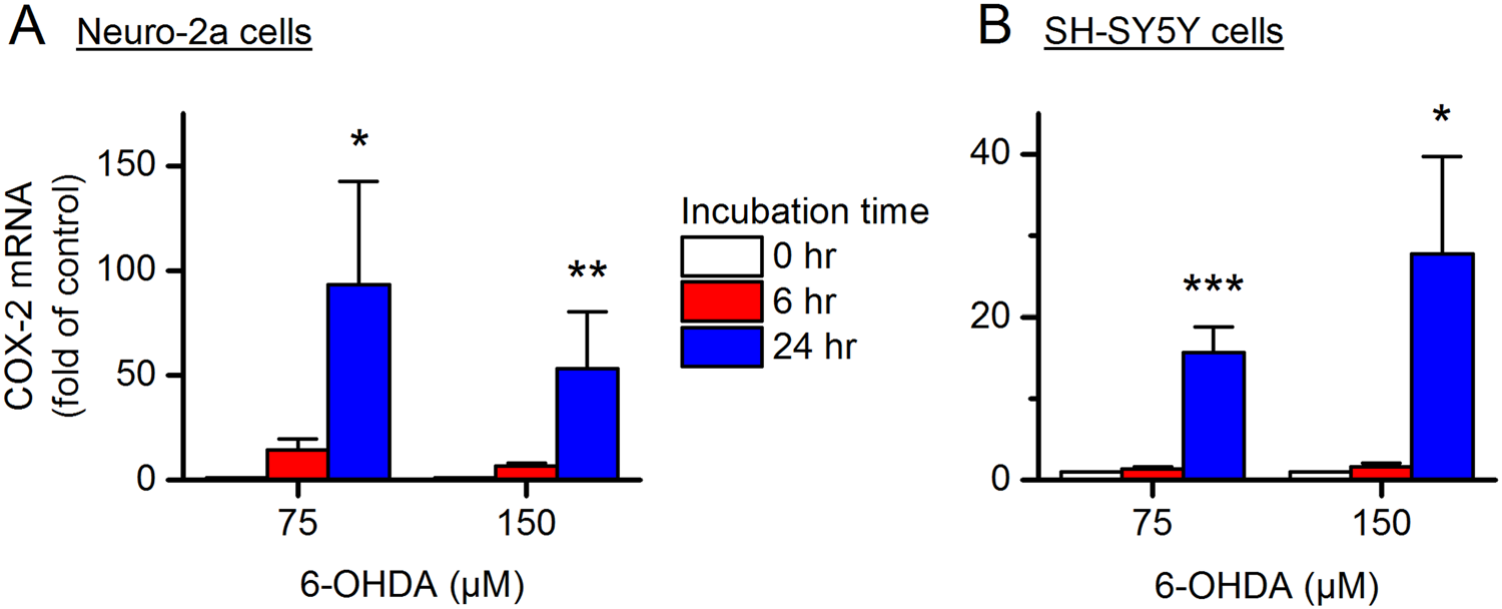
6-OHDA induces cyclooxygenase-2 (COX-2) expression. Neuro-2a cells (**A**) and SH-SY5Y cells (**B**) were treated with 6-OHDA at different concentrations (75 or 150 µM) for 0, 6 or 24 hr. COX-2 mRNA levels in these cells were measured by qPCR (*P < 0.05; **P < 0.01 compared to control, one-way ANOVA with *post-hoc* Dunnett’s Multiple Comparison Test). Data are presented as mean + SEM [*n* = 5–9 for Neuro-2a cells (**A**); *n* = 7 for SH-SY5Y cells (**B**)].

**Figure 4:**
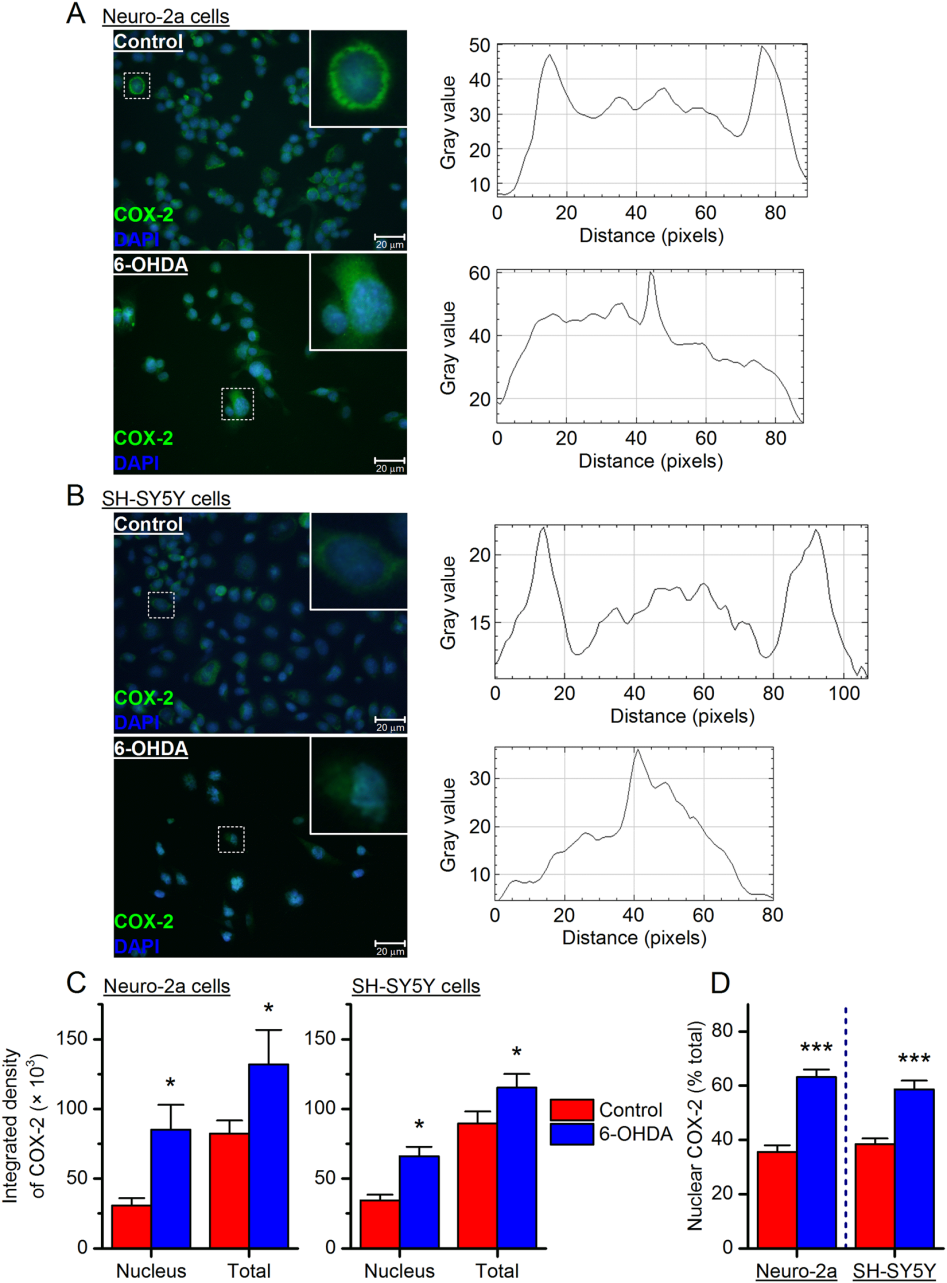
6-OHDA stimulation causes COX-2 translocation to the nucleus. Neuro-2a cells (**A**) and SH-SY5Y cells (**B**) were treated with 6-OHDA (75 and 150 µM, respectively) for 24 hr. Immunostaining was performed to show the expression of COX-2 protein (green) in these cells and the cell nuclei were visualized by DAPI staining (blue). The typical subcellular distribution of COX-2 protein in a single cell was quantified by measuring the fluorescence intensity along a line across the cell body using NIH ImageJ software. (**C**) The total and nuclear COX-2 in Neuro-2a cells (left) and SH-SY5Y cells (right) was quantified by measuring the integrated density of fluorescence with ImageJ (*P < 0.05 compared to control, one-way ANOVA with *post-hoc* Bonferroni’s Test). (**D**) The percentage of COX-2 protein in the nucleus was calculated in Neuro-2a cells (left) and SH-SY5Y cells (right) (***P < 0.001 compared to control, Student’s *t*-test). Data are presented as mean + SEM (*n* = 12–18). Scale bar = 20 µm.

The molecular mechanisms whereby 6-OHDA destroys dopaminergic neurons remain elusive, but it has been proposed that the neurotoxin enters the cells via dopamine transporter where it promotes chronic inflammatory processes and oxidative stress^21, 33, 34^. As a primary pro-inflammatory mediator, COX-2 is well known for its pathogenic roles in many chronic inflammation-associated conditions. To investigate the effect of 6-OHDA on COX-2 expression, we next measured COX-2 mRNA levels in the neurotoxin-treated cells using qPCR. We found that COX-2 was quickly and robustly induced in a time-dependent manner by 6-OHDA at both a low concentration (75 µM, ~EC_25_) and a high concentration (150 µM, ~EC_75_) in Neuro-2a cells (*P* < 0.05 and *P* < 0.01 for 24-hr incubation with 6-OHDA at 75 µM and 150 µM, respectively, Fig. 3A) and SH-SY5Y cells (*P* < 0.001 and *P* < 0.05 for 24-hr incubation with 75 µM and150 µM 6-OHDA, respectively, Fig. 3B). Immunochemistry further revealed that COX-2 was present at basal levels mainly in the cytosol of both Neuro-2a cells (Fig. 4A) and SH-SY5Y cells (Fig. 4B), where it synthesizes homeostatic prostanoids that are essential for many normal physiological functions. With 6-OHDA stimulation [75 µM for Neuro-2a cells and 150 µM for SH-SY5Y cells; concentrations were chosen based on their higher COX-2 induction (Fig. 3A and B)], COX-2 was remarkably induced in both the nucleus and cytosol of Neuro-2a and SH-SY5Y cells (*P* < 0.05, Fig. 4C). Interestingly, 6-OHDA-treated cells showed higher percentages of COX-2 expression in the nuclei when compared to vehicle-treated cells (*P* < 0.001, Fig. 4D), suggestive of a nuclear translocation of COX-2 triggered by 6-OHDA stimulation. Taken together, these results demonstrate that 6-OHDA treatment induces COX-2 activation characterized by expression induction and nuclear translocation in both Neuro-2a and SH-SY5Y cells.

### 6-OHDA treatment causes PGE_2_ biosynthesis and pro-inflammatory cytokine production

**Figure 5:**
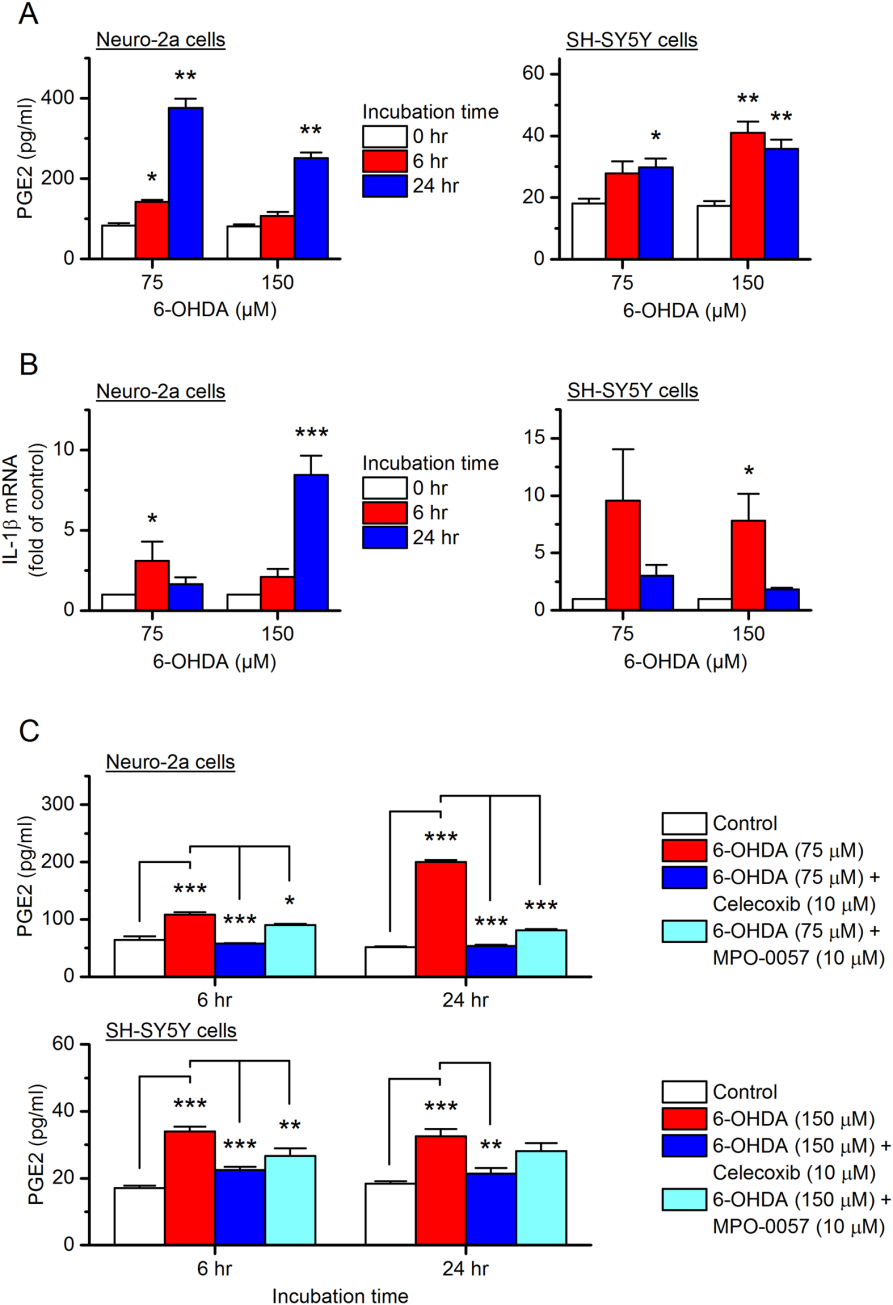
Effect of 6-OHDA on the syntheses of prostaglandin E2 (PGE_2_) and interleukine-1β (IL-1β). Neuro-2a cells (left) and SH-SY5Y cells (right) were treated with 6-OHDA (75 or 150 µM) for 0, 6 or 24 hr. (**A**) PGE_2_ levels in the culture medium of these cells were measured by ELISA (*P < 0.05; **P < 0.01 compared to control, one-way ANOVA with *post-hoc* Dunnett’s Multiple Comparison Test). (**B**) IL-1β mRNA levels within these cells were measured by qPCR (*P < 0.05; ***P < 0.001 compared to control, one-way ANOVA with *post-hoc* Dunnett’s Multiple Comparison Test). (**C**) Neuro-2a cells (top) and SH-SY5Y cells (bottom) were pretreated with selective COX-2 inhibitor celecoxib (10 µM) or microsomal prostaglandin E synthase-1 (mPGES-1) inhibitor MPO-0057 (or 8n, 10 µM) for 15 min, followed by 24-hr incubation with 6-OHDA (75 µM for Neuro-2a and 150 µM for SH-SY5Y cells). The PGE_2_ levels in culture medium were assessed by ELISA (*P < 0.05; **P < 0.01; ***P < 0.001 compared to control, one-way ANOVA with *post-hoc* Bonferroni’s Multiple Comparison Test for selected pairs as indicated). Data are presented as mean + SEM (*n* = 3–4).

To investigate the involvement of COX-2 in 6-OHDA-mediated inflammation and neurotoxicity, we next quantified the prostaglandins secreted into the culture medium from the neurotoxin-treated cells using ELISA. We found that the 6-OHDA stimulation substantially increased PGE_2_ in the culture medium by nearly 5-fold in Neuro-2a cells (*P* < 0.01 with 75 µM 6-OHDA incubation for 24 hr, Fig. 5A left) and 3-fold in SH-SY5Y cells (*P* < 0.01 with 150 µM 6-OHDA incubation for 24 hr, Fig. 5A right). Moreover, 6-OHDA treatment significantly upregulated the pro-inflammatory cytokine – interleukin-1β (IL-1β) within Neuro-2a cells (*P* < 0.05 with 75 µM 6-OHDA incubation for 6 hr; *P* < 0.001 with 150 µM 6-OHDA incubation for 24 hr, Fig. 5B left) and SH-SY5Y cells (*P* < 0.05 with 150 µM 6-OHDA incubation for 6 hr, Fig. 5B right), measured by qPCR for its mRNA levels.

Owing to their consistently high COX-2 induction (Fig. 3A and B) and PGE_2_ synthesis (Fig. 5A), we used 75 µM 6-OHDA to treat Neuro-2a cells and 150 µM 6-OHDA for SH-SY5Y cells throughout the rest of this study. To evaluate the contribution of COX-2 to 6-OHDA-induced PGE_2_ biosynthesis, the cells were pretreated with selective COX-2 inhibitor celecoxib (10 µM) for 15 min before a 6- or 24-hr incubation with 6-OHDA. Pretreatment with celecoxib significantly blocked the 6-OHDA-induced PGE_2_ secretion by both Neuro-2a cells (*P* < 0.001 with 75 µM 6-OHDA for both 6- and 24-hr incubation, Fig. 5C top) and SH-SY5Y cells (*P* < 0.001 and *P* < 0.01 with 150 µM 6-OHDA for 6-hr and 24-hr incubation, respectively, Fig. 5C bottom).

Prostaglandin E synthase (PGES) is the terminal enzyme for the PGE_2_ biosynthesis, and has three isoforms: microsomal prostaglandin E synthase-1 (mPGES-1 or PTGES), mPGES-2 (or PTGES2), and cytosolic PGES (cPGES or PTGES3). Among these three isozymes, mPGES-1 is functionally coupled to COX-2 and directly synthesizes PGE_2_ from COX-2-derived PGH_2_ in response to various detrimental stimuli^35^. We recently reported a novel selective mPGES-1 inhibitor MPO-0057 (or 8n)^36^, which was demonstrated to suppress lipopolysaccharide (LPS)-induced PGE_2_ synthesis in mouse macrophages^36^. Here, 6-OHDA-stimuated PGE_2_ synthesis was blocked by 10 µM compound MPO-0057 in both Neuro-2a cells (*P* < 0.05 and *P* < 0.001 with 75 µM 6-OHDA for 6-hr and 24-hr incubation, respectively, Fig. 5C top) and SH-SY5Y cells (*P* < 0.01 with 150 µM 6-OHDA for 6 hr-incubation, Fig. 5C bottom). These data together suggest that 6-OHDA promotes COX-2 activation in these two neuronal cell lines, leading to PGE_2_ synthesis and pro-inflammatory cytokine induction.

### PGE_2_ mediates cAMP signaling via EP2 receptor in Neuro-2a and SH-SY5Y cells

**Figure 6:**
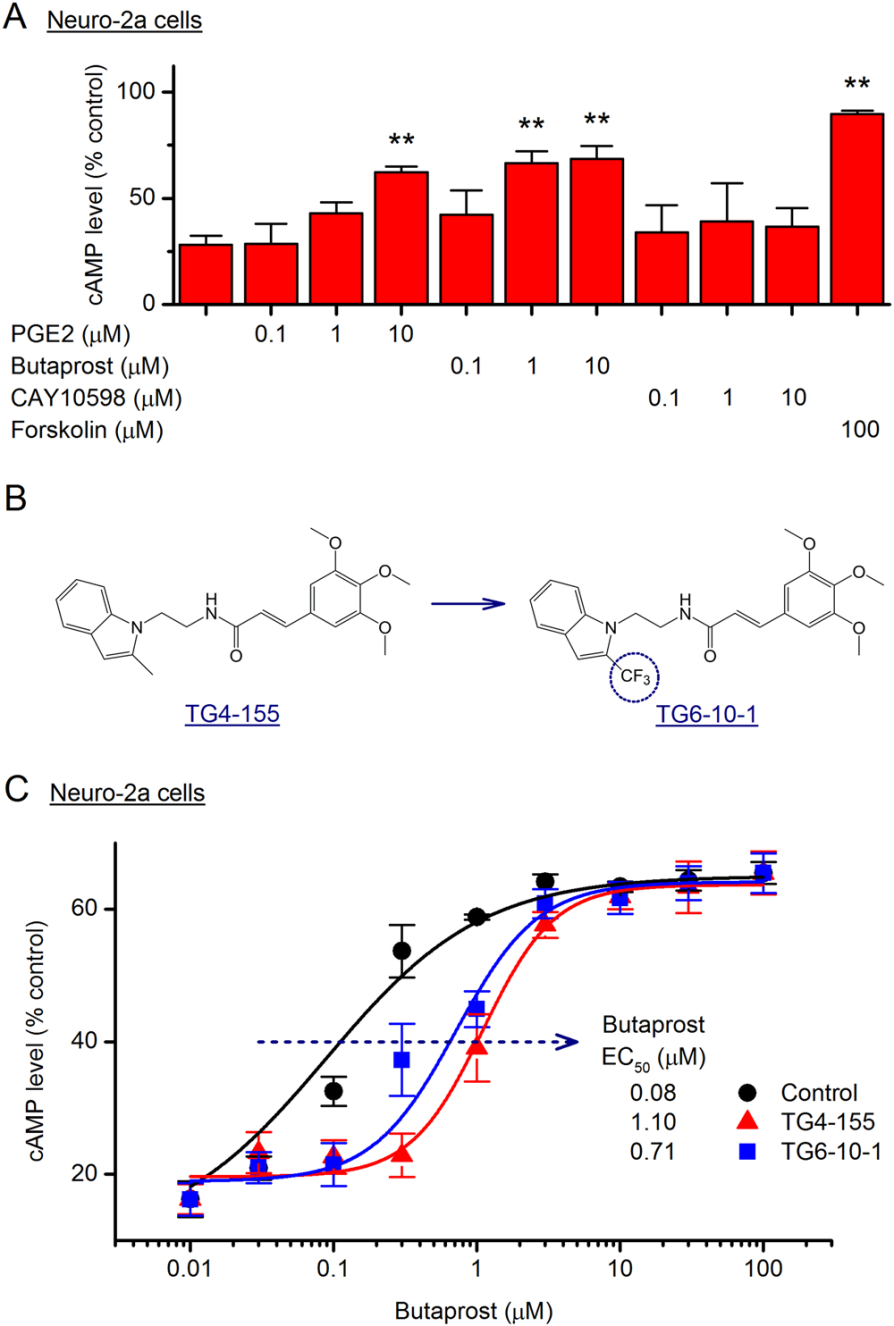
PGE_2_ mediates cAMP signaling in mouse Neuro-2a cells via EP2 receptor. (**A**) Neuro-2a cells were treated with PGE_2_ (0.1, 1 or 10 µM), EP2 selective agonist butaprost (0.1, 1 or 10 µM), EP4 selective agonist CAY10598 (0.1, 1 or 10 µM), or a direct activator of adenylyl cyclase forskolin (100 µM) for 40 min. The cAMP levels in the cells were measured by time-resolved fluorescence energy transfer (TR-FRET) assay (**P < 0.01 compared to control, one-way ANOVA with *post-hoc* Dunnett’s Multiple Comparison Test). Data are shown as mean + SEM (*n* = 5). (**B**) Chemical structures of novel selective EP2 antagonists TG4-155 and TG6-10-1. (**C**) The selective inhibition of compounds TG4-155 and TG6-10-1 (10 µM) on EP2 receptor in butaprost-treated Neuro-2a cells was evaluated by TR-FRET cAMP assay. The calculated butaprost EC_50_ was 80 nM and increased to 1100 and 710 nM in the presence of TG4-155 and TG6-10-1, respectively. Data are presented as mean ± SEM (*n* = 5).

PGE_2_ has four receptors EP1-EP4, among which EP2 and EP4 are Gα_s_-coupled and mediate cAMP-dependent pathways that are involved in PGE_2_-mediated chronic inflammation and neurodegeneration^1, 2, 12^. To investigate the PGE_2_/cAMP signaling in Neuro-2a cells, we treated the cells with increasing concentrations of PGE_2_, EP2 selective agonist butaprost, or EP4 selective agonist CAY10598. Forskolin, a direct activator of the adenylyl cyclase, was used to show the maximal capability of the cells to synthesize cAMP. The cytosol cAMP levels were measured by a time-resolved fluorescence energy transfer (TR-FRET) assay. As shown in the Fig. 6A, both PGE_2_ and Butaprost induced cAMP accumulation in Neuro-2a cells in a concentration-dependent manner (*P* < 0.01 for 10 µM PGE_2_, 1 or 10 µM butaprost). Particularly, 10 µM butaprost nearly peaked the cAMP levels in these cells when compared to positive treatment by 100 µM forskolin (*P* < 0.01). On the contrary, EP4 selective agonist CAY10598 merely slightly increased the cAMP levels in these cells at 10 µM (Fig. 6A), a concentration that should be able to fully activate the EP4 receptor^37^.

Using high-throughput screening (HTS) and subsequent medicinal chemistry for hit optimization, we developed a series of novel small-molecule compounds that are among the first-generation EP2 selective antagonists^38–44^. Compound TG6-10-1 was created by introducing a trifluoromethyl group in the methylindole ring of an HTS hit compound TG4-155, aiming to improve its pharmacodynamic and pharmacokinetic properties (Fig. 6B)^38^. Development of these novel compounds provides unprecedented opportunities to study this receptor through an inhibitory strategy^45^. Both compounds TG4-155 and TG6-10-1 showed robust inhibition on EP2 receptor in butaprost-treated Neuro-2a cells, demonstrated by an up to 10-fold rightward shift of butaprost dose-response curve when either compound (10 µM) was present (Fig. 6C).

**Figure 7:**
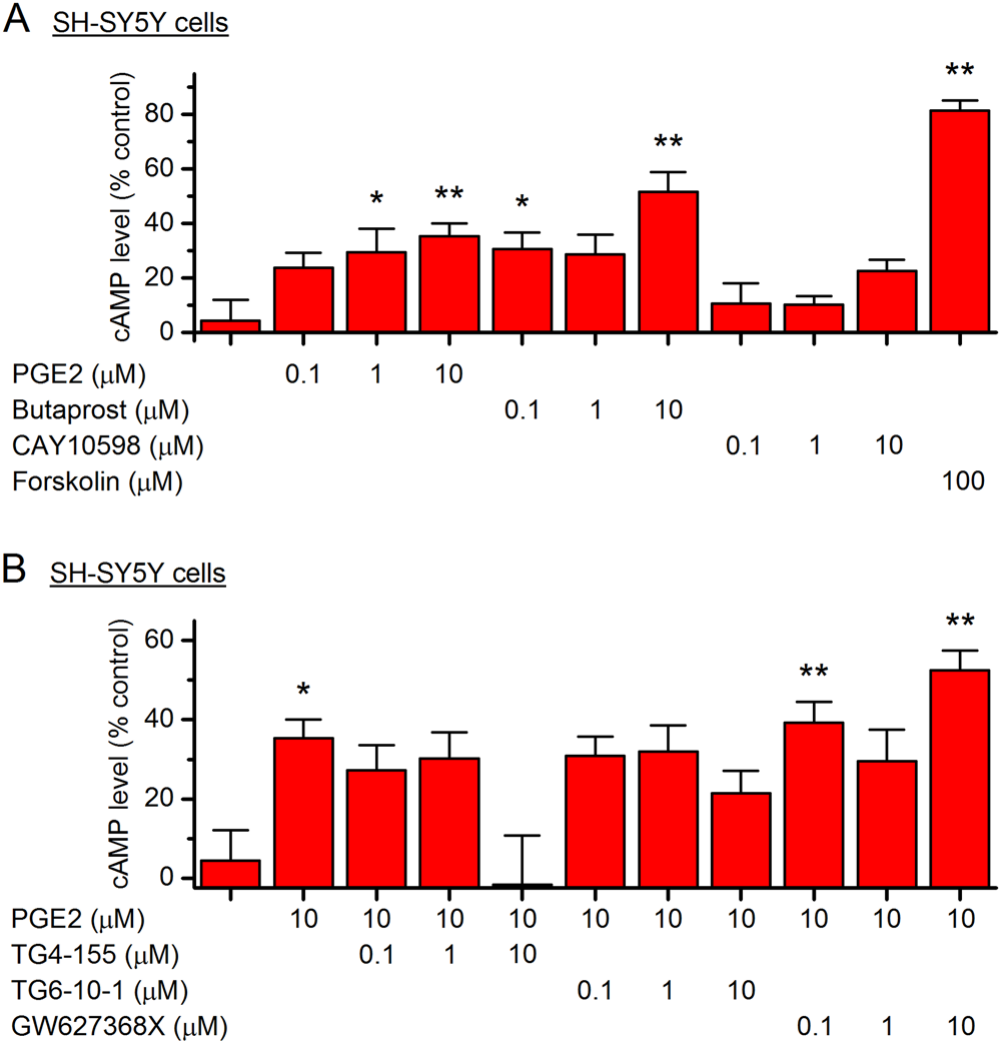
PGE_2_-mediated cAMP signaling in human SH-SY5Y cells. (**A**) SH-SY5Y cells were treated with PGE_2_ (0.1, 1 or 10 µM), butaprost (0.1, 1 or 10 µM), CAY10598 (0.1, 1 or 10 µM), or forskolin (100 µM) for 40 min. The cAMP concentrations in the cells were measured by TR-FRET assay (*P < 0.05; **P < 0.01 compared to control, one-way ANOVA with *post-hoc* Dunnett’s Multiple Comparison Test). (**B**) SH-SY5Y cells were treated with TG4-155 (0.1, 1 or 10 µM), TG6-10-1 (0.1, 1 or 10 µM), or EP4 antagonist GW627368X (0.1, 1 or 10 µM), followed by incubation with PGE_2_ (10 µM) for 40 min. The cAMP concentrations in the cells were measured by TR-FRET assay (*P < 0.05; **P < 0.01 compared to control, one-way ANOVA with *post-hoc* Dunnett’s Multiple Comparison Test). Data are shown as mean + SEM (*n* = 6).

Similarly, PGE_2_ and Butaprost, but not CAY10598 (up to 10 µM), recapitulated the forskolin-promoted cAMP production in the SH-SY5Y cells in a concentration-dependent manner (*P* < 0.05 for 1 µM PGE_2_ or 0.1 µM butaprost; *P* < 0.01 for 10 µM PGE_2_, 10 µM butaprost, or 100 µM forskolin, Fig. 7A). Furthermore, PGE_2_ (10 µM)-induced cAMP accumulation in SH-SY5Y cells was robustly blocked by TG4-155 or TG6-10-1 also in a concentration-dependent manner (Fig. 7B). In contrast, EP4 selective antagonist GW627368X had no significant inhibition on cAMP synthesis in these PGE_2_-treated cells at up to 10 µM (Fig. 7B), a concentration at which GW627368X should be able to completely shut down the EP4 receptor^46^. These results from TR-FRET cAMP assay suggest that EP2 is the dominant Gα_s_-coupled PGE_2_ receptor in both Neuro-2a and SH-SY5Y cells.

### EP2 receptor inhibition is neuroprotective in 6-OHDA-induced neuronal injury

**Figure 8:**
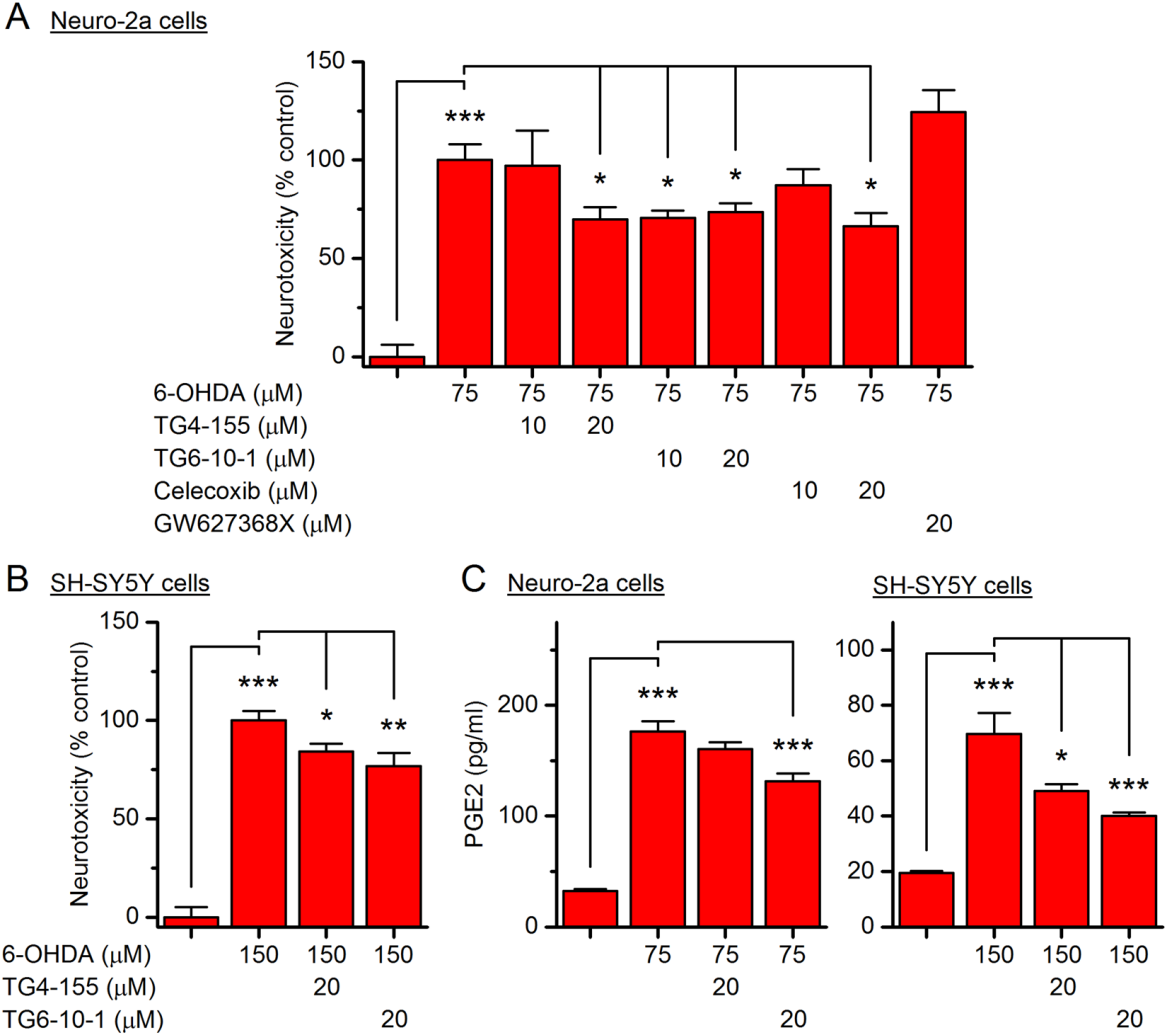
Selective inhibition on EP2 receptor reduces 6-OHDA-triggered neurotoxicity. (**A**) Neuro-2a cells were pretreated with TG4-155 (10 or 20 µM), TG6-10-1 (10 or 20 µM), celecoxib (10 or 20 µM), or GW627368X (20 µM) for 15 min, followed by treatment with 6-OHDA (75 µM). After 24 hr, the cell viability was measured by MTT assay (*P < 0.05; ***P < 0.001, one-way ANOVA with *post-hoc* Bonferroni’s Multiple Comparison Test for selected pairs as indicated). (**B**) SH-SY5Y cells were pretreated with TG4-155 (20 µM) or TG6-10-1 (20 µM) for 15 min, followed by treatment with 6-OHDA (150 µM) for 24 hr. The cell viability was measured by MTT assay (*P < 0.05; **P < 0.01; ***P < 0.001, one-way ANOVA with *post-hoc* Bonferroni’s Multiple Comparison Test for selected pairs as indicated). (**C**) Neuro-2a cells (left) and SH-SY5Y cells (right) were pretreated with TG4-155 (20 µM) or TG6-10-1 (20 µM) for 15 min, followed by 24-hr treatment with 6-OHDA (75 µM for Neuro-2a and 150 µM for SH-SY5Y cells). The PGE_2_ levels in culture medium were measured by ELISA (*P < 0.05; ***P < 0.001, one-way ANOVA with *post-hoc* Bonferroni’s Multiple Comparison Test for selected pairs as indicated). Data are shown as mean + SEM (*n* = 4–6).

We next examined whether PGE_2_/EP2 signaling is involved in the 6-OHDA-induced neuronal injury. To be consistent, we used 75 µM 6-OHDA to treat Neuro-2a cells and 150 µM 6-OHDA for SH-SY5Y cells. As shown in Fig. 8A, both EP2 antagonists TG4-155 and TG6-10-1 (10 or 20 µM) significantly reduced 6-OHDA (75 µM)-promoted cytotoxicity in Neuro-2a cells in a concentration-dependent manner, measured by MTT reduction assay 24 hr after 6-OHDA incubation (*P* < 0.05). This observation was completely recapitulated by selective COX-2 inhibitor celecoxib also in a concentration-dependent manner (*P* < 0.05, Fig. 8A), but not by EP4 selective antagonist GW627368X (20 µM) (Fig. 8A). Similarly, the 6-OHDA (150 µM)-promoted cytotoxicity in SH-SY5Y cells was also blocked by pretreatment with TG4-155 or TG6-10-1 (*P* < 0.05 for TG4-155; *P* < 0.01 for TG6-10-1, Fig. 8B). Furthermore, pretreatment with these two EP2 selective antagonists also decreased 6-OHDA-induced PGE_2_ secretion from Neuro-2a cells (*P* < 0.001 for TG6-10-1, Fig. 8C left) and SH-SY5Y cells (*P* < 0.05 for TG4-155; *P* < 0.001 for TG6-10-1, Fig. 8C right).

It appears that 10 µM TG4-155 showed remarkable inhibition on EP2-mediated cAMP production in these cells (Figs 6C and 7B), but merely moderately reduced the 6-OHDA-induced cytotoxicity (Fig. 8A and B). It should be noted that the cell-based cAMP assay was carried out in HBSS system requiring TG4-155 incubation for less than 1 hr, whereas the MTT cytotoxicity assay was performed 24 hr after the compound was added into the culture medium containing 10% FBS. It is likely that TG4-155 was more stable in HBSS than in culture medium, and thus showed more robust effect in the cAMP assay. Furthermore, TG6-10-1 showed slightly higher neuroprotection and inhibition on PGE_2_ synthesis than TG4-155 in both 6-OHDA-treated Neuro-2a and SH-SY5Y cells (Fig. 8A–C), although its potency of inhibiting EP2 receptor was considerably lower than that of TG4-155 (Figs 6C and 7B). This incongruity might also be explained by the structural stability of these two compounds. TG4-155 showed instant half-life (<10 min) in mouse or human liver microsomes, suggestive of its metabolic instability *in vitro* ^38^. In addition, TG4-155 had a relatively short plasma half-life (~0.6 hr) after systemic administration in mice^38^. With the introduction of fluorine (Fig. 6B), TG6-10-1 showed improved *in vitro* metabolic stability^38^, as well as longer plasma half-life in mice (~1.6–1.8 hr)^39, 41, 42^. Owing to its more stable chemical structure, TG6-10-1 might act on EP2 receptor longer than TG4-155 in the culture systems, thereby causing more protection of the 6-OHDA-treated cells and better inhibition on their PGE_2_ production, although both compounds were added into the culture medium 24 hr prior to the MTT assay. Nevertheless, these data together suggest that PGE_2_ signaling via EP2 receptor, but not EP4, contributes to 6-OHDA-induced cytotoxicity and PGE_2_ induction in Neuro-2a and SH-SY5Y cells.

## Discussion

In the present study, we demonstrated that stimulation with 6-OHDA rapidly caused COX-2 activation – expression upregulation and nuclear translocation – in both mouse Neuro-2a and human SH-SY5Y cells, leading to pro-inflammatory reaction characterized by PGE_2_ synthesis and cytokine (IL-1β) induction. We also found that PGE_2_ mediated cAMP-dependent pathways in these cells mainly through EP2 receptor subtype. The finding that 6-OHDA-induced cytotoxicity in these NeuN- and TH-positive cells was largely blocked by pharmacological inhibition on EP2 receptor, but not EP4 receptor, sheds light on the COX-2 downstream signaling pathway that is involved in the neurotoxin-mediated neuronal inflammation and injury.

6-OHDA is a commonly used neurotoxin to induce the neuronal loss in animal PD models; however, the molecular mechanisms underlying the progressive death of dopaminergic cells in the SNpc are not fully understood. Here we presented evidence that COX-2 is induced by 6-OHDA in both mouse and human neuronal cell lines that express TH (Fig. 3A and B), leading to PGE_2_ synthesis (Fig. 5A and C) and IL-1β secretion (Fig. 5B). Therefore, 6-OHDA-provoked inflammatory processes in these cells are – at least partially – mediated by COX-2 and prostaglandin cascade. Upregulated COX-2 has been reported in many neurological disorders including stroke^6^, epilepsy^39, 47, 48^, and neurodegenerative diseases^49, 50^, and has been proposed to essentially contribute to the neuronal injury via initiating pro-inflammatory processes and imposing oxidative stress on the neurons^51, 52^. COX-2 is selectively induced in SNpc dopaminergic neurons in 1-methyl-4-phenyl-1,2,3,6-tetrahydropyridine (MPTP)-administered mice^53^, and in 6-OHDA-treated rats^54^. The genetic ablation or pharmacological inhibition of COX-2 is protective for the TH neurons and relieves the oxidative stress in these animals^53, 55^. Epidemiological studies over the past decades also suggest that the chronic use of non-steroidal anti-inflammatory drugs (NSAIDs) might be associated with reduced incidence of PD^56–58^. These preclinical and clinical data suggest an essential role for COX-2 in the neurodegenerative process of PD. However, the conception of COX-2 as a therapeutic target has been dampened over the past decade due to the growing recognition of adverse effects of several COX-2 inhibitor drugs on the cardiovascular and cerebrovascular systems^59^, inspiring us that the downstream prostaglandin signaling pathways might provide alternative targets for therapeutics with more specificity.

In response to external stimuli such as injury, infection and inflammation, COX-2 converts the cell membrane-released arachidonic acids to PGH_2_ in the cytoplasm where the unstable intermediate prostaglandins are further catalyzed to prostanoids by tissue-specific isomerases^1^. Unexpectedly, we found translocation of COX-2 from the cytoplasm to nucleus in the 6-OHDA-treated Neuro-2a and SH-SY5Y cells (Fig. 4A–D). The translocation of COX-2 between the nucleus and cytosol has been reported in IL-1β-treated vascular endothelial cells^60^, retinal Müller cells after hypoxia^61^, human breast carcinoma^62^, and bladder cancer cells^63^, but not in brain neurons prior to this study. COX-2 activity has been linked to several nuclear receptors and transcription factors such as peroxisome proliferator-activated receptors (PPARs), octamer-binding transcription factor 4 (Oct-4), and nuclear factor-κB (NF-κB)^63–66^. Whether the nuclear translocation of COX-2 in these two neuroblastoma cell lines would indicate a novel function of COX-2 in the transcriptional regulation of genes that are associated with 6-OHDA-promoted oxidative stress and apoptosis needs further investigation.

EP2 and EP4 are the two currently known PGE_2_ receptor subtypes that are coupled to Gα_s_ and mediate cAMP-dependent signaling pathways in both brain neurons and glia^2, 12^. PGE_2_ signaling via the EP2 receptor leads to α-synuclein aggregate-mediated neurotoxicity in mouse MPTP model of PD^18^. Moreover, EP2 receptor activation facilitates microglial and astrocytic inflammatory responses to MPTP and causes loss of dopaminergic neurons in the SNpc^19^, suggesting an exacerbating role of EP2 receptor in the pathogenesis of PD. Conversely, pharmacological activation of EP4 receptor has been reported to prevent the MPTP-induced loss of SNpc neurons, whereas the genetic ablation of the receptor aggravated MPTP-associated pro-inflammatory processes^20^. These previously findings together suggest that EP2 and EP4 might function oppositely during the degenerative progression in PD or other neurodegenerative diseases^12, 67–69^. Consistently, we showed that the inhibition on EP2 receptor – but not EP4 – significantly reduced 6-OHDA-triggered neuronal death (Fig. 8A and B). In fact, EP4 receptor inhibition by GW627368X showed a trend of exacerbating the 6-OHDA-mediated cytotoxicity (Fig. 8A).

COX-2 inhibition by celecoxib completely blocked 6-OHDA-induced PGE_2_ synthesis in both Neuro-2a and SH-SY5Y cells (Fig. 5C). However, highly COX-2-selective celecoxib – at a relatively high concentration (up to 20 µM) which would completely shut down the enzymatic activity of COX-2 – was only able to block up to 35% cytotoxicity in these 6-OHDA-treated cells (Fig. 8A), suggesting that some COX-2/PGE_2_-independent mechanisms might also be involved in 6-OHDA-mediated neurotoxicity. Nevertheless, the EP2 antagonists afforded a similar level of neuroprotection to that celecoxib provided following 6-OHDA treatment (Fig. 8A), insinuating that the COX-2-mediated injury in these cells can mostly be attributed to EP2 receptor activation by elevated PGE_2_. However, the contribution of Gα_q_-coupled EP1 and Gα_i_-coupled EP3 receptors to the 6-OHDA-induced neurotoxicity cannot be excluded in this study, as PGE_2_ presumably also activates these two EP receptors following 6-OHDA-triggered COX-2 induction. Also interestingly, EP2 receptor activation by butaprost has been reported to be neuroprotective in rat midbrain cultures containing approximately 5% dopaminergic neurons^70^. These seemingly differing results [see also in *N*-Methyl-D-aspartate (NMDA)-induced excitotoxicity in rat primary cortical cultures^71–74^] are not unexpected, given that EP2 is expressed in both glial cells and neurons^71, 75^, and whether its activation is beneficial or detrimental might be determined by its cell type-specific expression^2^. Therefore, whether these selective EP2 antagonists can provide sufficient beneficial effects on SNpc neurons following MPTP or 6-OHDA treatment *in vivo* remains an important topic for the future study.

## Materials and Methods

### Cell culture

To ensure the identity of the cells, the mouse neuroblastoma cell line – Neuro-2a and the human neuroblastoma cell line - SH-SY5Y were directly purchased from the American Type Culture Collection (ATCC^®^: #CCL-131™ and #CRL-2266™, respectively) (Manassas, VA, USA). The Neuro-2a cells were cultured in Dulbecco’s Modified Eagle Medium (DMEM) supplemental with 10% fetal bovine serum (FBS), 1% MEM non-essential amino acids solution, 100 U/ml penicillin and 100 µg/ml streptomycin (Corning Life Sciences, Corning, NY, USA) at 37 °C in a humidified atmosphere consisting of 5% CO_2_/95% air. The SH-SY5Y cells were maintained in 50% ATCC-formulated Eagle’s Minimum Essential Medium (EMEM) + 50% Ham’s F-12 medium (Corning Life Sciences) supplemental with 10% FBS, 100 U/ml penicillin and 100 µg/ml streptomycin.

### Chemicals and reagents

PGE_2_, butaprost, TG4-155, CAY10598 and GW627368X were purchased from Cayman Chemical (Ann Arbor, MI, USA). 6-hydroxydopamine (6-OHDA), forskolin and rolipram were purchased from Sigma-Aldrich (St. Louis, MO, USA). Compound TG6-10-1 was from MedChem Express (Monmouth Junction, NJ, USA). The selectivity and potency of TG4-155 and TG6-10-1 were evaluated blindly and compared between batches for consistency as described previously^38^.

### Immunocytochemistry

Cells were plated onto Poly-D-Lysine coated 24-well plates. After each treatment, cells were fixed with 4% paraformaldehyde (PFA) (Affymetrix, Santa Clara, CA) in PBS, followed by permeation with 0.2% Triton X-100 (Thermo Fisher Scientific, Waltham, MA, USA) in PBS. After incubation in blocking solution – 10% horse serum (Sigma-Aldrich, St. Louis, MO, USA) in PBS for 2 hr, the cells were incubated with primary antibodies: NeuN (EMD Millipore, #MAB377), tyrosine hydroxylase (TH) (Cell Signaling Technology, #2792), or COX-2 (abcam, #ab15191) overnight, and followed by staining with Alexa Fluor^®^ 488 or 546-conjugated secondary antibodies (Invitrogen, Carlsbad, CA, USA) for 2 hr and DAPI (10 µg/ml in PBS, Invitrogen) for 10 min. Slides were mounted using DPX mountant (Electron Microscopy Sciences, Hatfield, PA, USA). Images were obtained using EVOS FL Auto Cell Imaging System (Invitrogen). The fluorescence intensity was quantified using ImageJ software developed at the National Institutes of Health (NIH).

### Cell viability assay

Cell viability was measured using the Vybrant MTT [3-(4,5-dimethylthiazol-2-yl)-2,5-diphenyltetrazolium bromide] reduction assay kit (Invitrogen) as previously described^45^. In brief, cells were cultured in 96-well plates (5,000 cells/well). After treatment, MTT was added into each well at a final concentration of 0.5 mg/ml and the cells were incubated at 37 °C for 4 hr. Living cells converted MTT to insoluble formazan, which was dissolved in DMSO. Absorbance of the formazan was measured by a microplate reader (Molecular Devices, Sunnyvale, CA, USA) at 540 nm with a reference wavelength at 630 nm. The dose-response curves were generated and EC_50_ values were calculated using OriginPro software (OriginLab, Northampton, MA, USA).

### Quantitative PCR

mRNA levels of interested genes were quantified by quantitative PCR (qPCR) as described previously^76, 77^. In brief, total RNA was isolated using Trizol (Invitrogen) with the PureLink RNA mini kit (Qiagen, Hilden, Germany) from cultured cells. RNA concentration and purity were measured by a NanoDrop spectrophotometer (Thermo Fisher Scientific). First-strand complementary DNA (cDNA) synthesis was performed with 1 µg of total RNA, using SuperScript III One-Step RT-PCR System (Invitrogen) according to manufacturer’s guidelines. 1.5 µl of cDNA was used to set up each 20-µl qPCR reaction mixture following SYBR Green PCR Master Mix manual (Thermo Fisher Scientific). Real-time PCR was performed using the StepOnePlus Real-Time PCR System (Applied Biosystems, Foster City, CA, USA). Cycling conditions were as follows: 95 °C for 2 min followed by 40 cycles of 95 °C for 15 s and then 60 °C for 1 min. Melting curve analysis was used to verify single-species PCR product. Fluorescent data were acquired at the 60 °C step. The cycle thresholds for GAPDH was used as an internal control for relative quantification. Samples without cDNA template served as the negative controls. Primers used for qPCR were listed in Table 1. The mRNA level of an interested gene was normalized to the control in each experiment to limit unwanted sources of variation.

**Table 1:** Sequences of the primers for qPCR.

Gene	Forward primer	Reverse primer	Accession number
**Mouse**			
COX-2	5′-CTCCACCGCCACCACTAC-3′	5′-TGGATTGGAACAGCAAGGAT-3′	NM_011198.3
IL-1β	5′-TGAGCACCTTCTTTTCCTTCA-3′	5′-TTGTCTAATGGGAACGTCACAC-3′	NM_008361.3
GAPDH	5′-TGTCCGTCGTGGATCTGAC-3′	5′-CCTGCTTCACCACCTTCTTG-3′	NM_008084.2
**Human**			
COX-2	5′-GGTCTGGTGCCTGGTCTGAT-3′	5′-TCCTGTTTAAGCACATCGCATACT-3′	NM_000963.3
IL-1β	5′-TACCTGTCCTGCGTGTTGAA-3′	5′-TCTTTGGGTAATTTTTGGGATCT-3′	NM_000576.2
GAPDH	5′-GTCAAGGCTGAGAACGGGAA-3'	5′-AAATGAGCCCCAGCCTTCTC-3′	NM_002046.5

### PGE_2_ measurement

The PGE_2_ levels in culture medium were measured by ELISA kit from Arbor Assays (Ann Arbor, MI, USA). In brief, cells were cultured in 24-well plates with 90,000 cells/well for Neuro-2a and 150,000 cells/well for SH-SY5Y cells. After each treatment, 50 µl medium was taken for PGE_2_ measurement according to the manufacturer’s protocol. The optical density generated from each well was measured in a microplate reader (Molecular Devices) at 450 nm. A standard curve for PGE_2_ was run within each experiment.

### Cell-based TR-FRET cAMP assay

Cytosol cAMP was measured with a cell-based homogeneous time-resolved fluorescence resonance energy transfer (TR-FRET) method (Cisbio Bioassays, Codolet, France) as described previously^45, 71, 75^. The assay is based on generation of a strong FRET signal upon the interaction of two molecules: an anti-cAMP antibody coupled to a FRET donor (cryptate) and cAMP coupled to a FRET acceptor (d2). Endogenous cAMP produced by cells competes with labeled cAMP for binding to the cAMP antibody and thus reduces the FRET signal. Cells were seeded into 384-well plates in 30 µl complete medium (4,000 cells/well) and grown overnight. The medium was carefully withdrawn and 10 µl Hank’s Buffered Salt Solution (HBSS) (Hyclone, Logan, Utah, USA) plus 20 µM rolipram was added into the wells to block phosphodiesterase. The cells were incubated at room temperature for 30 min, and then treated with vehicle or tested compound for 5–10 min before addition of EP agonists. The cells were further incubated at room temperature for 40 min, then lysed in 10 µl lysis buffer containing the FRET acceptor cAMP-d2 and 1 min later another 10 µl lysis buffer with anti-cAMP-cryptate was added. After 60–90 min incubation at room temperature, the FRET signal was measured by an Envision 2103 Multilabel Plate Reader (PerkinElmer, Waltham, MA, USA) with excitation at 340 nm and dual emissions at 665 nm and 590 nm for d2 and cryptate (100 µs delay), respectively. The FRET signal was expressed as: F665/F590 × 10^4^ and normalized to the controls to indicate the cAMP levels. The dose-response curves were generated and EC_50_ values were calculated using OriginPro software (OriginLab).

### Statistical analysis

Statistical analyses were performed using Prism (GraphPad Software, La Jolla, CA, USA) by one-way ANOVA with *post-hoc* Bonferroni/Dunnett’s test for multiple comparisons or Student’s *t*-test as appropriate. *P* < 0.05 was considered to be statistically significant. All data are presented as mean + or ± SEM.
